# 3D Extension at Plate Boundaries Accommodated by Interacting Fault Systems

**DOI:** 10.1038/s41598-020-65599-5

**Published:** 2020-05-26

**Authors:** Luca Collanega, Giacomo Corti, Anna Breda, Matteo Massironi, Derek Keir

**Affiliations:** 1 Dipartimento di Geoscienze, Università degli Studi di Padova, Via G. Gradenigo 6, 35131 Padova, Italy; 2 Consiglio Nazionale delle Ricerche (CNR), Istituto di Geoscienze e Georisorse (IGG), UOS Firenze, Via G. la Pira 4, 50121 Firenze, Italy; 3 Dipartimento di Scienze della Terra, Università degli Studi di Firenze, Via G. la Pira 4, 50121 Firenze, Italy; 4 School of Ocean and Earth Science, University of Southampton, Southampton, SO14 3ZH UK

**Keywords:** Tectonics, Geodynamics, Structural geology

## Abstract

Complex patterns of normal faults with multiple orientations and/or highly curved shapes have been traditionally explained by successive tectonic phases of 2-dimensional deformation. Alternatively, multiple fault sets have been proposed to develop simultaneously and in orthorhombic symmetry during a single phase of 3-dimensional deformation. We use analogue models of normal faults to demonstrate that, without the influence of pre-existing structures, 3D extension is preferentially accommodated by the alternate, rather than simultaneous, development of faults with different trends. By means of stress-driven interactions, 3D deformation can be partitioned into coupled systems of normal faults, which display geometries commonly observed in tectonic settings affected by interacting plate boundaries. Under radial extension, deformation is accommodated by major curvilinear grabens coupled with minor perpendicular faults, resulting in the triple junctions of grabens observed in Afar. On the other hand, the alternate development of perpendicular faults accommodates synchronous bi-directional and mutually perpendicular extension, giving the same fault pattern observed in the Barents Sea rift-shear margin.

## Introduction

The deformation of the crust has long been explained by the Andersonian model of faulting^1^, which predicts the development of conjugate faults striking in map view perpendicular to the extension direction^2–4^. Since no deformation occurs parallel to the faults, this theory effectively assumes the deformation to be bi-dimensional (2D). Whereas this classic model requires successive phases of tectonic deformation to account for multiple sets of non-parallel structures^5,6^, three-dimensional (3D) deformation may readily explain the development of these complex fault patterns by using a single tectonic phase (i.e. polymodal faulting^7,8^). On the grounds of theoretical arguments^9,10^, field observations^9^ and rock mechanics experiments^11^, Reches demonstrated orthorhombic patterns of normal faults (i.e four sets of faults symmetrically arranged with respect to the principal strain axes) to be the preferential fault arrangement to accommodate 3D strain fields. However, obliquely convergent and divergent margins (e.g. Central California, the Northumberland Basin in northern Britain, the Dead Sea Basin), where the deformation is necessarily three-dimensional, are characterised by complex combinations of strike-slip faults, thrusts and normal faults^7,12–14^, suggesting that bulk 3D strain is “kinematically partitioned” into strike slip-, extension- and shortening-dominated regions^15^. Understanding how 3D deformation can be accommodated is central to the correct interpretation of fault geometries, and thus to the reconstruction of tectonic histories, the prediction of sub-seismic fracture networks and subsurface fluid circulation, and the evaluation of earthquake hazard.

The orthorhombic fault patterns predicted by Reches^9^ derive from the hypothesis of a homogeneous stress/strain distribution, which implies that faults should develop symmetrically with respect to the principal stress/strain axes^9,10^. The theory considers the entire fault network to develop instantaneously, thereby assuming that the deformation occurs under a temporally stable stress/strain field. However, in light of seismological studies, a temporally and spatially uniform stress field is unrealistic in an actively deforming system, with post-earthquake variations of the seismicity rates clearly demonstrating an immediate stress-drop following the rupture of a fault^16–18^. By perturbing their local stress fields (and thus also the fluid circulation), adjacent faults can influence one another during their development, establishing fault interactions and feeding a self-organisation process of fault patterns (sensu Cowie^19^). Although these concepts can successfully explain fault distribution in 2D strain fields^20,21^ and the evolution of 3D networks of small-scale faults and fractures^22^ (offset <5 m, width of few centimeters), they have not yet been applied to explain the development of km-scale faults under a 3D strain field.

To assess fault interactions and organisational patterns in a 3D deforming system, we used simple, gravity-driven, ductile-brittle analogue models. We reproduced symmetrical 3D strain fields, which (i) may be representative of the deformation occurring at symmetric intersections of different plate boundaries (e.g. triple junctions, areas of rift-plume interaction, intersections of rift segments) and (ii) are suitable to highlight any local perturbation of the stress field induced by fault activity. Finally, we compared final fault geometries (i) between our model of isotropic radial extension and the Afar triple junction, where the East African, Gulf of Aden and Red Sea rifts intersect at approximately 120°, and (ii) between our model of synchronous bidirectional extension and the Barents Sea rift-strike slip margin, where the Atlantic and Arctic rifts intersect at approximately 90°. Our work shows how well-developed faults can constrain the local strain field, forcing new faults to develop perpendicular to them. Hence, a regional 3D strain field can be accommodated by systems of interacting fault sets, which strike perpendicularly to the local extension direction, and thus in accordance with Anderson’s theory of faulting^1^.

## Results

To reproduce isotropic radial extension and bidirectional extension, we constrained the lateral flow of the basal ductile layer of the models, and thus the strain field, by creating a circular and a square model respectively (see Methods for details on model materials and scaling). In order to determine diagnostic fault geometries of true 3D strain fields with respect to polyphasic extension, we reproduced both synchronous bidirectional extension (i.e. simultaneous extension along two perpendicular directions) and 2-phase bidirectional extension (i.e. two successive phases of uniaxial extension perpendicular one to the other). Our models reproduce extension of a brittle layer (1 cm-thick) overlying a ductile layer (1.5 cm-thick), representing possibly different tectonic environments^23^. Although the models were specifically scaled to simulate extension of an uppermost brittle layer (1–3 km-thick) overlying a lithological interval (3–4.5 km-thick) with ductile rheology in the shallow crust, they may be also representative of the whole upper crust (10–15 km-thick) overlying the ductile lower crust (15–22.5 km-thick; see Methods for details on the scaling procedure). The geometrical scaling ratio, combined with the viscosity of the ductile material and the velocity of deformation of the models, may correspond to a natural velocity in the range of ∼2.9–29 mm/yr, which correlates well with natural extensional systems (see Methods section). Hence, the geometries of our models are representative of the normal faults patterns developing in a natural rift system (e.g. the Barents Sea, Afar Triple Junction).

### Isotropic radial extension

**Figure 1:**
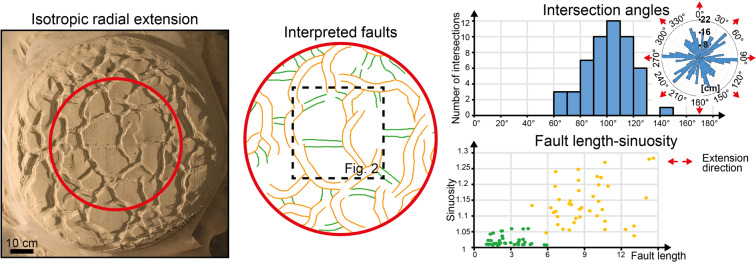
Final fault geometries following isotropic radial extension. Major curvilinear faults (orange segments) intersect minor straight faults (green segments) in their points of maximum curvature. In the fault length-sinuosity diagram, orange points and green points correspond to curvilinear and straight faults, respectively. See Methods for details on how we took measurement of strike distribution, fault length and sinuosity, and intersection angles between faults.

Isotropic radial extension results in a pattern of polygonal faults with a rather uniform strike distribution (Fig. 1, Rose diagram). Normal faults generally intersect each other at high angles (90°–120°), and are arranged in triple junctions of grabens (Fig. 1, Intersection angles). Two arms of the triple junctions typically accrue more displacement than the third one, forming a major curvilinear graben. Based on their length and sinuosity (see Methods for details on the measurement procedure), faults can therefore be subdivided into two fault populations (Fig. 1, Fault length-sinuosity): (i) major, high-sinuosity normal faults (orange segments in Fig. 1, Interpreted faults) and (ii) minor, straight faults (green segments in Fig. 1, Interpreted faults). The straight faults generally intersect the high-sinuosity faults in the points of maximum curvature of the latter and terminate against them, producing Y-shaped intersections.

**Figure 2:**
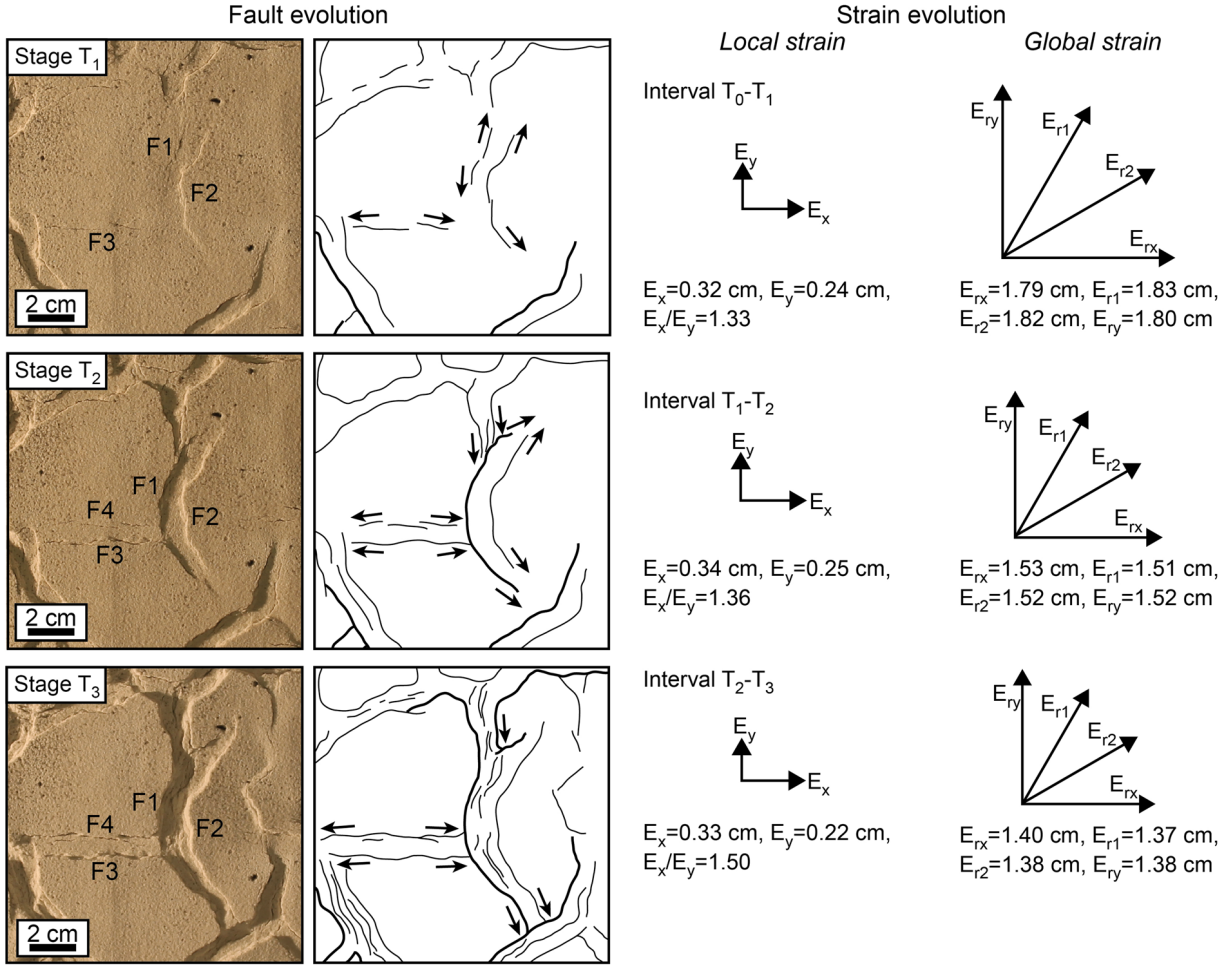
Fault evolution under isotropic radial extension. The vectors E_x_, E_y_, E_r_ indicate the amount of extension along the x, y, and the radial directions. Extension on the x direction appears dominant within the area shown in the boxes on the left-hand side, whilst at the global scale extension is approximately equal along each radial direction.

The major curvilinear graben (F1, F2) and the associated minor normal faults (F3) develop simultaneously since the onset of deformation (Fig. 2, Stage T_1_), suggesting these structures are kinematically related although they are not physically connected yet. As the deformation proceeds, the major and minor grabens propagate laterally (Fig. 2, Stage T_2_) until they intersect, producing triple junctions of grabens (Fig. 2, Stage T_3_). During the whole experiment, the curvilinear graben accrue more displacement than the straight faults, reflecting an anisotropic strain field with a preferential extension direction perpendicular to the curvilinear faults (Fig. 2, Local strain). Notably, the local strain field associated with each triple junction is highly anisotropic, despite the global strain field of the whole model being radially isotropic throughout the experiment (Fig. 2, Global strain). Hence, the anisotropic local strain fields associated with differently oriented triple junctions appear to compensate one another, suggesting an interaction between adjacent faults.

### Bidirectional extension

**Figure 3:**
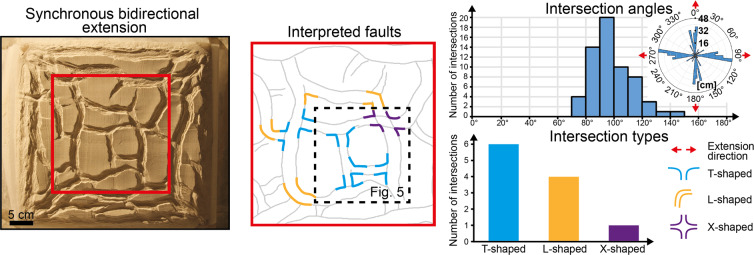
Final fault geometries following synchronous bidirectional extension along two perpendicular directions. Faults intersect each other approximately at 90°, producing preferentially T-shaped and L-shaped intersections. See Methods for details on how we took measurement of fault strike distribution and intersection angles.

**Figure 4:**
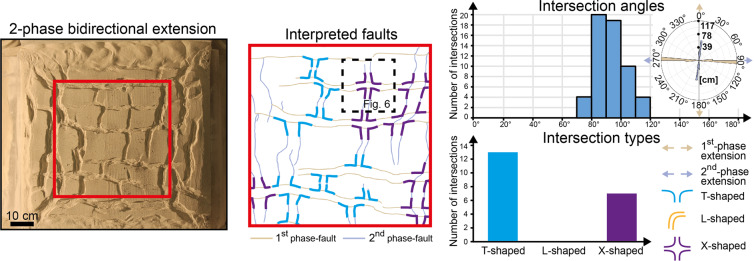
Final fault geometries following 2-phase bidirectional extension. The model has been first deformed by applying N-S extension, then by applying E-W extension. Second-phase faults systematically terminate against and occasionally crosscut first-phase faults, producing T-shaped and X-shaped intersections. See Methods for details on how we took measurement of fault strike distribution and intersection angles.

When a model is extended simultaneously along two perpendicular directions, a distinct fault system develops perpendicular to each extension direction, as indicated by the development of N-S-striking and E-W-striking faults (Fig. 3). Faults of each system terminate against faults of the other in equal measure, resulting (i) in the two fault systems being equally developed in terms of total length (Fig. 3, Rose diagram) and (ii) in the development of T-shaped and L-shaped intersections oriented in all possible ways (i.e. along both the N-S and E-W axes of the model; Fig. 3, Interpreted faults). The geometry of the fault intersections is controlled by the relative maturity of the intersecting faults, with T-shaped intersections developing when incipient faults meet more mature faults and L-shaped intersections developing between faults with similar maturity. In contrast, when two different phases of uniaxial extension are applied, the geometry of the fault intersections is mainly controlled by the relative timing of the two fault systems, with second-phase faults systematically terminating against first-phase faults (Fig. 4). As a consequence, in 2-phase extension models, the dominant intersection style is represented by T-shaped intersections all oriented along the same axis of the model, although there are also some X-shaped intersections (Fig. 4, Intersection types). Since second-phase faults generally terminate against first-phase faults, second-phase faults are less developed than first-phase faults both in terms of total length (total length of first-phase faults = 176 cm; total length of second-phase faults = 136 cm; Fig. 4, Rose diagram) and average length (average length of first-phase faults = 12.57 cm, average length of second-phase faults = 4.39 cm).

**Figure 5:**
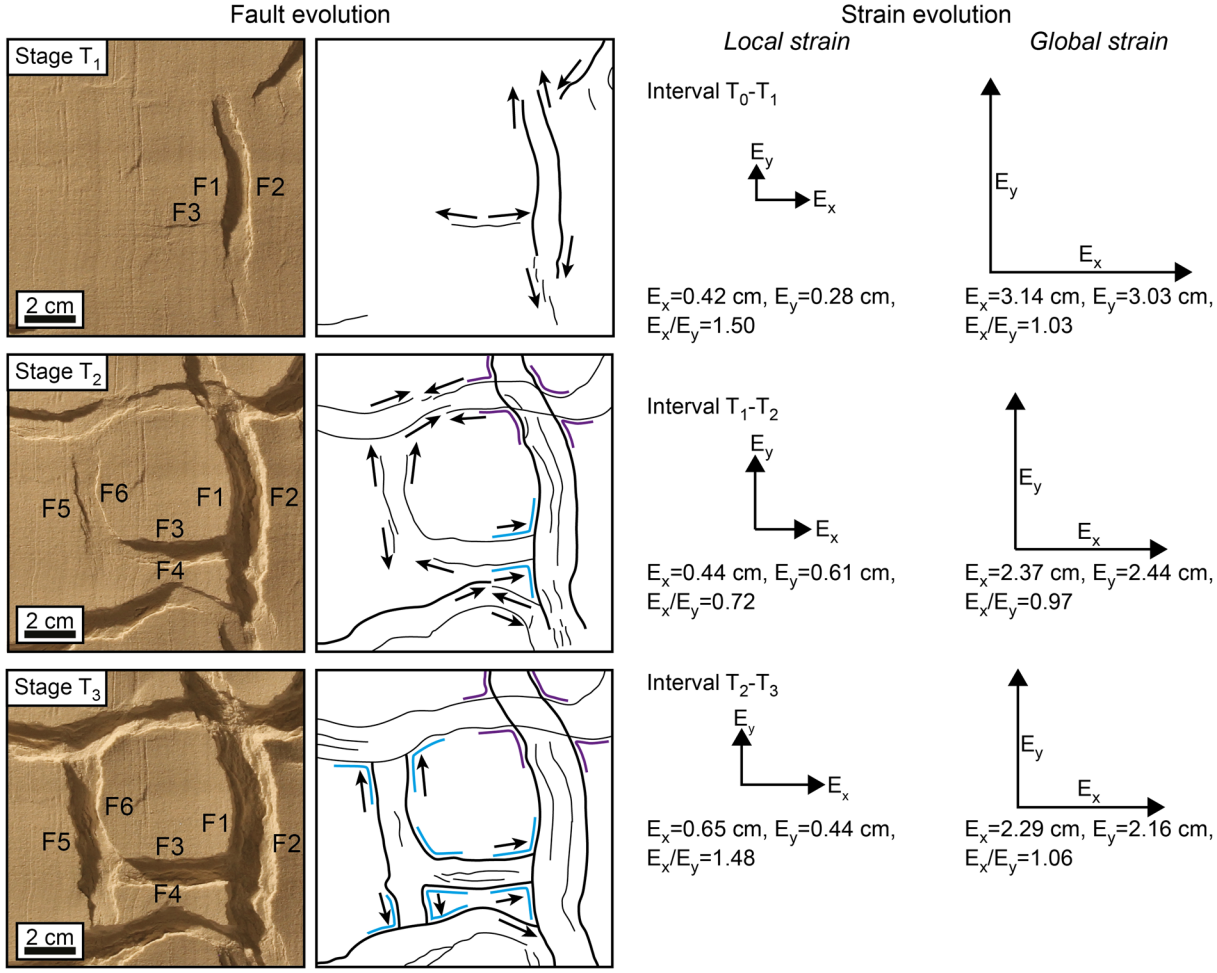
Fault evolution under synchronous bidirectional extension. The vectors E_x_ and E_y_ indicate the amount of extension along the x and y directions, at the local and global scales. The alternate development of perpendicular faults reflects the oscillation of the dominant extension direction at the local scale, which contrasts with a symmetrical and stable global strain field.

**Figure 6:**
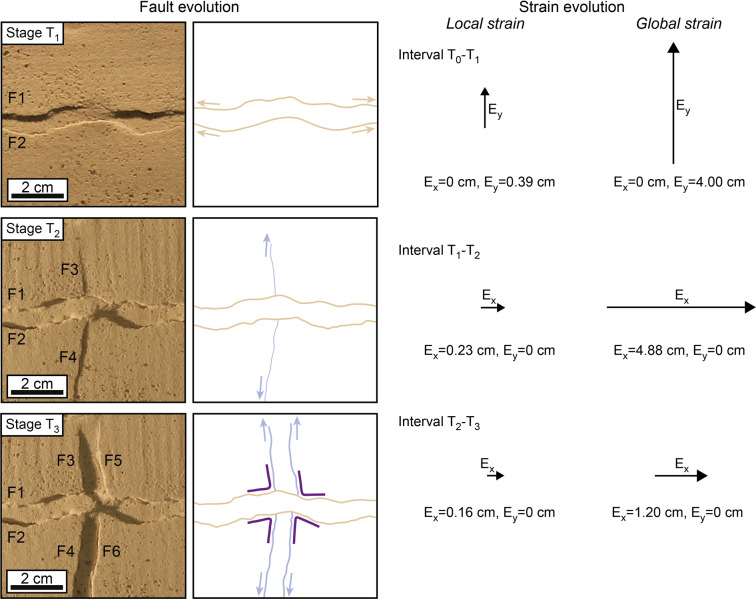
Fault evolution during two phases of uniaxial and mutually perpendicular extension. The vectors E_x_ and E_y_ indicate the amount of extension along the x and y directions, at the local and global scales. Second-phase faults initially terminate against first-phase faults (Stage T_2_), crosscutting them with increasing amount of deformation (Stage T_3_).

In the model of synchronous bidirectional extension, the very similar geometrical properties and the completely equivalent intersecting relationships of E-W-striking and N-S-striking faults reflect the strongly interrelated development of the two fault systems (Fig. 5), with faults of one system influencing faults of the other to the same extent. Indeed, the development of a fault perpendicular to an extension direction is generally accompanied shortly afterwards by the nucleation of a new fault perpendicular to the other extension direction within a close distance, as shown by the nucleation of F3 next to F1, F2 (Fig. 5; Stage T_1_) and by the nucleation of F5, F6 next to F3, F4 (Fig. 5; Stage T_2_). In contrast, when the two extension directions are applied during two different phases, a major influence of first-phase (i.e. E-W-striking) faults on second-phase (i.e. N-S-striking) faults is apparent both in terms of geometries and intersecting relationships. On the one hand, first-phase faults acted as mechanical barriers^5^, which restricted the lateral propagation of second-phase faults (resulting in the lower average and total length of second-phase faults with respect to first-phase faults); on the other hand, first-phase faults offered ideal nucleation sites for second-phase normal faults^6^ (e.g. F3 nucleating from F1; Fig. 6, Stage T_2_). Indeed, more fault segments initiated during the second extension phase (n = 31) with respect to the first extension phase (n = 14), despite being less developed in terms of total length.

With increasing amount of deformation, originally isolated second-phase faults may merge together to form long N-S striking faults, which crosscut the pre-existing E-W striking faults, producing X-shaped intersections (cf. Stage T_2_ and Stage T_3_ in Fig. 6). In contrast, X-shaped intersections are very rare in the model of synchronous bidirectional extension (one X-shaped intersection out of eleven; Fig. 3, Intersection types), most likely due to the transfer of displacement between simultaneously active faults, which prevented one fault from crosscutting the other.

### Radial extension in the afar depression

**Figure 7:**
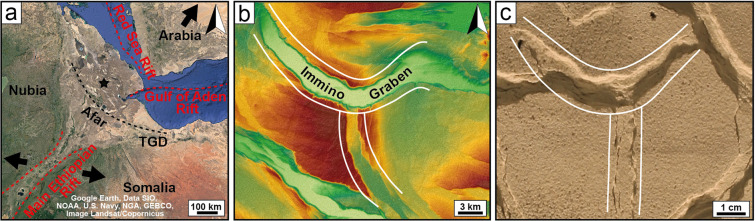
Evidence for radial extension in the Afar Depression. (**a**) Location of Afar at the intersection between the Red Sea, Gulf of Aden and East African rifts. Relative motion between Nubia and Somalia taken from Corti^70^ and relative motion of Arabia with respect to Nubia taken from McClusky^71^ (Map data: Google, Data SIO, NOAA, U.S: Navy, NGA, GEBCO, Image Landsat/Copernicus). TGD, Tendaho Goba’ad Discontinuity. The black star indicates the location of the study area in (**b)**. (**b, c**) Similarity between fault geometries in a digital elevation model (map data: NASA JPL. *NASA Shuttle Radar Topography Mission Global 1 arc second*. 2013, distributed by NASA EOSDIS Land Processes DAAC; visualised in ArcGIS 10.5.1) and in our analogue model (**c**). In both cases, a major curvilinear graben is intersected in its point of maximum curvature by a minor normal straight graben.

The Afar Depression has a spatially and temporally complex strain field due to its position at the triple junction between the Gulf of Aden, Red Sea and East African rifts^24–26^ (Fig. 7a). At the present day, two main structural domains can be distinguished, with NE-SW-directed Africa-Arabia extension to the north and ESE-WNW-directed Nubia-Somalia extension to the south^27^ (Fig. 7a). These regions are separated by the Tendaho Goba’ad Discontinuity (TGD), across which most of the mutually perpendicular extension is thought to be partitioned. However, the combination of structural trends coupled with direct geodetic measurements of ground motion suggest that not all the extension is partitioned and that significant portions of Afar extend in more than one direction^28,29^. In particular, the intrusion of a ~10 km long dike striking WNW-ESE shows that the southern portion of Afar in the Ethiopian rift experiences some NE-SW directed extension best explained by Africa-Arabia motion, confirming the kinematic models based on Afar-wide GPS station^30,31^. This extension is roughly perpendicular to the dominant near ~E-W extension across the Ethiopian rift, suggesting that extension directions associated with the different rift systems can act simultaneously in the same area.

In light of our model of radial extension, the complex pattern of curvilinear faults of central Afar can be interpreted as the result of a partially radial strain field. In particular, 100 km north of the Tendaho Goba’ad Discontinuity, a major, approximately WNW-ESE-striking curvilinear graben (the Immino Graben) intersects at its point of maximum curvature with a less developed, approximately N-S-striking graben, displaying a strong similarity with the triple junctions of grabens observed in our model (Fig. 6b, c). The development of this coupled system of perpendicular grabens is likely to reflect the onset of a radial strain field, although the fact that the major curvilinear graben is oriented approximately WNW-ESE suggests a dominant influence of the NE-SW Africa-Arabia motion. The radial component of the strain field is likely to result from the interaction between the three intersecting rift systems, with the associated extension directions acting simultaneously.

### Synchronous bidirectional extension in the Barents Sea rift-shear margin

**Figure 8:**
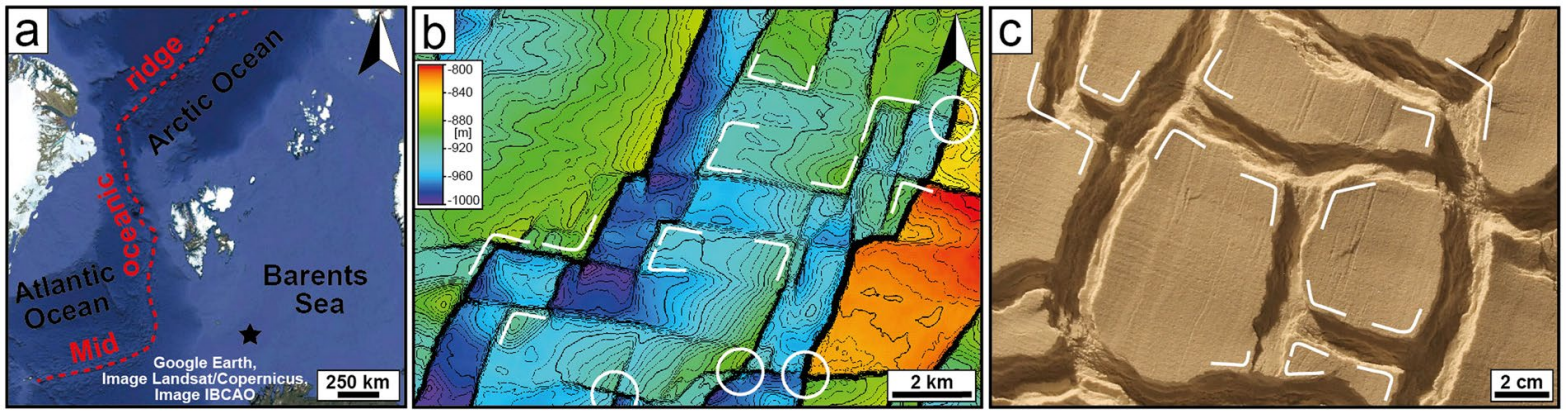
Evidence for synchronous bidirectional extension in the Barents Sea. (**a**) Location of the Barents along the rift-strike slip margin between the Atlantic and the Arctic oceans (Map data: Google, Image Landsat/Copernicus, Image IBCAO). The black star indicates the location of the study area in (**b). (b, c)**, Similarity between fault geometries on a 3D seismic surface (map created in Petrel 2018.2) (**b**) and in our analogue model (**c**) In both cases, two approximately perpendicular fault systems abut against each other, producing preferentially T-shaped and L-shaped intersections. However, in the natural example (**b**) WNW-ESE and NNE-SSW faults occasionally crosscut each other, producing X-shaped intersections (indicated by the white circles).

The Barents Sea is a rift-strike slip margin resulting from the interaction between the Atlantic and Arctic rifts^32,33^ (Fig. 8a), whose rifting and later break-up occurred largely simultaneously during the late Mesozoic-Cenozoic^32^. During this time interval, two nearly perpendicular fault systems developed at a regional scale, with WNW-ESE^34–36^ and N-S to NNE-SSW faults^35,37^ suggested to be the response to the opening of the Arctic and the Atlantic rifts respectively^38^. As rifting progressed, these fault systems were reactivated several times^37^ with phases of activity varying in different sectors of the Barents Platform^36–38^, indicating temporal and spatial variations of the main extension direction. Such instability of the stress field has been suggested to reflect a complex interplay between the Atlantic and the Arctic rifting, with multiple tectonic pulses^38^.

Three-dimensional seismic data from a shallow-depth area of the Barents Sea reveal that WNW-ESE and NNE-SSW faults have very similar geometries to those of our experiment on synchronous bidirectional extension (Fig. 8b, c). In particular, we note that the predominant intersection style between faults is represented by L-shaped and T-shaped intersections oriented both ways, which are diagnostic features of synchronous bidirectional extension instead of 2-phase extension (cf. Figs. 3 and 4). We thus suggest that the extension directions of the Atlantic and the Arctic rifting, at least periodically, acted simultaneously producing a 3D strain field with a synchronous bidirectional extension. As extensional activity has been recorded on both the fault systems^35,38^, deformation does not seem to be partitioned into its extensional and strike-slip components, with faults developing under a completely extensional strain field. As rifting proceeded, (i) increasing influence of the Atlantic rift^35,38^ and (ii) reactivation of deep, N-S-striking structures resulted in the extension being localised solely on the NNE-SSW faults^35^; whereas the WNW-ESE faults are likely to have accommodated the strike-slip component of the deformation. Consequently, NNE-SSW faults may have propagated laterally and crosscut the WNW-ESE faults (Fig. 8b, white circles), partially obliterating the original intersection style.

## Discussion

Our models of radial extension and synchronous bidirectional extension highlight a strong spatial and temporal instability of 3D extensional strain fields, which were traditionally modelled as homogeneous and stable^9,10^. The fault evolution in our models suggests that the instability of 3D strain fields is governed by the interactions between adjacent structures mediated by local perturbation of the stress field, which have been previously shown to be fundamental constraints on 2D deformation^21,39,40^. It is well-known that in 2D strain fields the local stress drop due to the development of a fault hinders the nucleation^20^ and propagation^21^ of new parallel – but not co-linear – faults, resulting in distinctive spatial distribution^21^ and displacement profiles^41,42^. Similarly, in our models of 3D deformation, the development of a fault hinders the nucleation of faults with the same strike but, interestingly, it triggers the development of perpendicular ones. A similar process has been observed for new faults propagating outwards from reactivated structures, which strike perpendicular to the pre-existing structures rather than to the regional extension direction^5,43,44^. This coupled development of perpendicular faults (Fig. 5) suggests that the stress drop associated with the development of a new fault (or to the reactivation of a pre-existing structure) is localised to near the fault and has a “direction-sensitive” nature. By means of these “direction-sensitive” stress drops, mature faults control the strike of the incipient, adjacent faults, resulting into a “self-organisation process” of the fault pattern (sensu Cowie^19^).

Our models show that 3D extension of a rheologically homogeneous layer can be accommodated by systems of interacting normal faults. However, in the presence of pre-existing structures or pronounced variations of the mechanical stratigraphy, 3D deformation may be more easily accommodated by “strain partitioning” into domains subject to prevailing strike-slip, extensional or compressional kinematics^45–47^. Hence, our models appear to be well-representative of (i) the deformation occurring above a lithological interval with ductile rheology, and thus decoupled from the influence of deep, inherited structures, (ii) the well-distributed deformation in early-rifts, prior to the strain localisation along few, major structures^47,48^. The similarity between our models and natural examples suggests that (i) in the Afar triple junction, the interaction of three different rifts combined into radial extension (Fig. 7), and (ii) in the Barents Sea, the two extension directions of the Arctic and the Atlantic rifts resulted into synchronous bidirectional extension (Fig. 8). In particular, our model describes well the deformation pattern during the initial phase of the Barents Sea rifting. However, as rifting proceeded, deformation localised on the reactivated structures^35^, resulting into partitioning of the deformation between the NNE-SSW faults (extensional component) and the WNW-ESE (strike-slip component).

In conclusion, the concept of “direction-sensitive” stress drop can explain how faults can self-organise themselves in a 3D deforming system, so that a mature fault can actively control the strike of the new faults nucleating in its vicinity. Hence, we demonstrate that, without the influence of pre-existing structures, 3D strain fields can be accommodated by interacting systems of normal faults that strike perpendicular to each other. As slip on a mature fault locally rotates the extension direction, each individual fault experiences 2D deformation in accord with Anderson’s theory, although the overall fault network deforms in 3D. As such, our model shows how a 3D strain field can be partitioned into several interacting 2D strain fields, thereby bridging the gap between the theories of 2D and 3D strain.

## Methods

**Figure 9:**
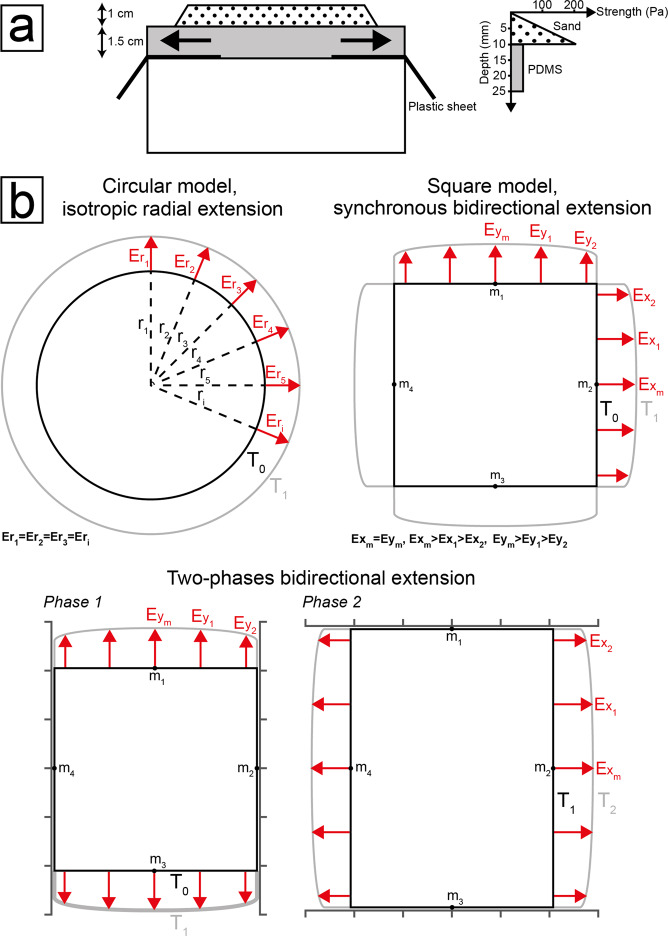
Modelling set-ups. (**a**,) schematic cross-section of the models and corresponding strength profile. (**b**,) schematic top-views of the models in the three configurations investigated (the radius of the circular model at time T_0_ is 46 cm, the side of the square model at time T_0_ is 81 cm). Red arrows indicate the extension at the model boundaries (the length of the arrows has been exaggerated to highlight the evolution of the model). The natural tendency of PDMS to flow perpendicular to the model boundaries has been exploited to reproduce different strain fields.

To reproduce a 3D strain field, it is necessary that at least two extension directions act simultaneously on a model. In 2D deformation experiments, the extension is generally achieved by introducing velocity discontinuities through moving basal plates, thereby localising deformation and affecting the stress field in a dominant way^49,50^. Such localisation of deformation represents a major challenge when attempting to reproduce a 3D strain field, as it favours the onset of distinct areas subject either to one extension direction or to the other but not to both of them. To overcome this issue, we used gravity-driven extension experiments^50–52^ (Fig. 9), where extension is achieved by the gravitational spreading of a basal ductile layer without introducing velocity discontinuities, which can localise the deformation. With this type of experimental set-up, we constructed simple, two-layer brittle-ductile models. We reproduced an upper brittle layer by using a mixture of quartz and K-feldspar sand (70:30% in weight), with a grain size <250 µm, angle of internal friction of ~39°, cohesion of ~65 Pa and density of ~1.55 g/cm^3^ (as measured by Montanari^53^). This brittle layer overlies a ductile layer made of transparent silicone (Polydimetilsiloxane, PDMS), characterized by Newtonian viscous rheology^54^ under the experimental conditions (i.e. low strain rate), with a viscosity of 2·10^4^ Pa·s and density of 0.96 g/cm^3^.

The models were designed with a geometric scale ratio such that 1 cm corresponds to 1–3 km in nature, with the aim of representing the development of normal faults within a 1.5–4.5 km brittle layer overlying a ductile level with a similar thickness. For a density of natural upper crustal materials of 2600–2700 kg m^−3^ (resulting in a density model to nature ratio ρ* of ∼0.6, where the asterisk denotes the model to nature ratio), a gravity ratio of 1 (the gravity is the same in nature and experiments) and the above mentioned geometric scaling ratio (h*∼3 ×1 0^−6^–1 × 10^−5^), the resulting model to nature stress ratio σ* = *ρ* * *g* * h* ^55–57^ amounts to ∼2 × 10^−6^ − 6 × 10^−6^. The frictional properties of the sand mixture used in the experiments scale well to the brittle rheology of the upper crust^58–61^: the angle of internal friction corresponds to that of most of the rocks (*ca*. 30°) and its cohesion scales properly to natural values (given that the model to nature cohesion ratio τ_0_* equates the model to nature average stress ratio σ* = 4 × 10^−6^, the resulting natural cohesion is τ_0n_ = τ_0m_/σ* = 1.6 × 10^7^).

For the ductile layer, the viscosity (*η*) of the PDMS at room temperature (2 × 10^4^ Pa·s) and the average velocity of deformation in the models (*v* = 10 mm/hr) can be scaled down considering the relation σ* = η*v*/h* ^55–57^. According to this, a natural viscosity of ∼10^19^ Pa·s for the ductile lithological interval^54,62^ corresponds to a natural velocity in the range ∼2.9–29 mm/yr, similar to natural extensional systems (cf. extension rate of 2–6 mm/yr for the East African Rift^63^, extension rate up to 23 mm/yr for the Gulf of Aden and Red Sea rifts^64^). Although the scaling to the uppermost crust is supported by the natural examples under consideration, our experiments can be also scaled to the whole continental crust. In this case, by considering a geometric scaling ratio such that 2.5 cm of the model correspond to 30 km in nature (i.e. a geometric scaling ratio h* = 8.3 × 10^−7^), the brittle layer in the model corresponds to a 12 km-thick upper crust overlying a 18 km-thick lower crust. By considering h*, the resulting model to nature stress ratio is σ* = 5 × 10^−7^. This, together with a natural viscosity of 10^21^ Pa·s for the lower crust gives a new viscosity ratio η* of 2 × 10^−17^, and results in a scaled natural velocity of deformation of 4.2 mm/yr, which is in line with several extensional settings, as outlined above.

Thanks to the fact that PDMS tends to flow perpendicular (i.e. along the shortest path) to the model boundaries, it was possible to reproduce different strain fields by creating models with different shapes. In particular, we created (i) a circular model, which extended equally along each radial direction, giving an isotropic radial strain field and (ii) a square model, which equally extended perpendicular to each model side, with the onset of synchronous bidirectional extension (Fig. 9). To keep the strain field stable during the experiments, we cut regularly PDMS along the edges of the models, preserving their original shapes. Although this type of experimental set-up allows deformation of the models without introducing any velocity discontinuity, it gives no direct control on the applied stress field, preventing us from taking direct measurement of the stress. In particular, a gradient of extension is likely to arise along the sides of the square model (with maximum extension at the mid points and minimum extension at the vertices; Fig. 9) due to the tendency of PDMS to develop a circular shape (as a result of the surface tension of fluids). However, the development of straight grabens parallel to the model sides (Figs. 3 and 4) implies that the extension gradient, or at least its effect on the fault geometries, was negligible in the central part of the model, which is the base for all the considerations made in this work.

To identify diagnostic geometries of synchronous bidirectional extension, we reproduced also 2-phase uniaxial extension in models having the same shape, size and mechanical layering as the previous ones, and we compared the final fault patterns. By putting lateral barriers on two opposite sides of the model, first we forced the lateral flow of PDMS along the N-S direction (first deformation phase); then, perpendicular to it, along an E-W direction (second deformation phase; Fig. 9). At the onset of the second deformation phase, the lower ductile level of PDMS was already thinned by the first extension phase, meaning different experimental conditions during the two deformation phases. As the deformation of the model is driven by the gravitational collapse of the PDMS layer, a thinner level of PDMS results in a lower deformation rate, slowing down the propagation of the deformation from the boundaries towards the centre of the model. To get the same amount of first-phase and second-phase extension in the central part of the model, thus we had to leave the second extension direction acting for a longer time than the first extension direction (2 h30 m vs 1 h15 m). Hence, at the global scale, the magnitude of second-phase extension (E_y_ = 6.08 cm) is higher than the first one (E_x_ = 4 cm) but, within the central part of the model, the magnitudes of first- and second-phase extension are comparable (cf. local strain in Stage T_1_ and cumulative local strain in Stage T_2_ and T_3_, Fig. 6).

All the models are characterised by the development of conjugate systems of faults arranged in symmetrical grabens, reflecting the fact that a symmetrical stress field was applied to a laterally homogeneous model^51,52^. These grabens are approximately evenly spaced, exhibiting approximately the same lateral spacing (ca. 7 cm) and width (ca. 1.5 cm) in all the models, which is most likely controlled by the relative thickness of the brittle and ductile layers^65,66^. Although systems of equally distributed symmetrical grabens are quite rare in nature, they represent a simple, and thus ideal, setting to highlight the interaction between developing faults. To quantitatively characterise the fault geometries, we calculated the total length, the average length and the intersection angles for each experiment. Length-weighted rose diagrams have been used to represent the fault strike, and histograms have been used to represent the distribution of the intersection angles (for intersection angle, we took the angle having its vertex in the fault intersections and defined by two points taken 6.9 cm away from the vertex on both the intersecting segments). In the models of synchronous and 2-phase extension, we distinguished different intersecting relationships: (i) X-shaped intersections, where two faults crosscut each other, (ii) T-shaped intersections, where a fault terminate against another fault and (iii) L-shaped intersections, where two faults terminate one against the other. Since radial extension models display a sole intersection style with Y-shaped intersections between two populations of faults, we found that the resulting final fault geometries could be better described by using fault length and sinuosity. Fault length was calculated by measuring the along strike distance between the nodes of the faults; then, fault length was divided by the rectilinear length (i.e. distance between the end points of the faults along a straight line), getting an estimate of the fault sinuosity. To record the evolution of the faults, we took high-resolution photographs of the top of the models (ground pixel size of 0.025 cm) every two minutes. By measuring the variation of distance between markers on the model surface, we obtained an estimate of the extension along specific directions. As fault geometries provide clear evidence for the interactions between developing faults (which is the goal of the current work), we did not perform any inversion of the stress field^67–69^, assuming the stress axes to correspond with the strain axes.
